# Middle aortic syndrome in children and adolescents

**DOI:** 10.21542/gcsp.2022.20

**Published:** 2022-12-30

**Authors:** Mustafa Musajee, Marisa Gasparini, Douglas J. Stewart, Narayan Karunanithy, Manish D. Sinha, Morad Sallam

**Affiliations:** 1 Department of Vascular Surgery, Guy’s and St Thomas’ NHS Foundation Trust, London, United Kingdom; 2 School of Biomedical Engineering and Imaging Sciences, King’s College London, London, United Kingdom; 3 Department of Paediatric Nephrology, Evelina London Children’s Hospital, Guy’s & St Thomas’ NHS Foundation Trust, London, United Kingdom; 4 Kings College London, London, United Kingdom

## Abstract

Middle aortic syndrome is a rare form of renovascular disease that may present with severe hypertension during childhood. Narrowing of the abdominal aorta is often associated with narrowing of the renal and/or other visceral arteries and may be secondary to specific genetic syndromes. Following the optimization of blood pressure control, significant aortic narrowing often requires invasive management, including endovascular and surgical intervention. In younger children, endovascular therapy may be attempted in the first instance to acutely reduce the pressure gradient across the narrowing; however, a sustained benefit is rare. Once the child has grown to accommodate a graft of an adequate size, surgical therapy is indicated for patients in whom medical and/or endovascular management has not resulted in adequate blood pressure control. It is critical that individuals with middle aortic syndrome be managed by an experienced multidisciplinary team that includes medical, endovascular, and surgical expertise that can provide long-term care to monitor for recurrent hypertension and evidence of end-organ damage.

## Introduction

Middle aortic syndrome (MAS) is a rare condition characterized by segmental or diffuse narrowing of the abdominal and/or distal descending thoracic aorta, accompanied by varying degrees of involvement of the renal and visceral arteries^1, 2^. This disorder was first described in 1963 when Sen et al. presented a case series of 16 patients with stenosis of the middle aorta with an underlying inflammatory process identified on histology^3^. MAS is an important and significant cause of renovascular hypertension in children 1. Awareness of this condition is therefore crucial to identify new cases among hypertensive children, as interventions are available that may help improve blood pressure control and reduce the burden of the associated morbidity.

## Epidemiology

The term MAS is currently used to describe obstructive lesions of the aorta regardless of their aetiology. It accounts for 0.5–2% of all aortic narrowing cases^4^. The clinical features of MAS often manifest before 18 years of age. Younger children are more likely to have extensive and severe vessel involvement^5^. The mean age at diagnosis has reduced from 14.3 years to 7.1 years over the last two decades as a result of improved diagnostic capabilities using non-invasive modalities such as computed tomography angiography (CTA) and magnetic resonance angiography (MRA)^1, 6^. Although neither is indicated in the initial stages of hypertension work-up in children, the 2016 European Society of Hypertension guidelines for the management of high blood pressure in children recommend screening with renal Doppler ultrasound, which may detect evidence of renovascular disease, prompting further assessment with CTA or MRA^7^.

MAS has a similar incidence in males and females^1, 6^. The youngest reported case of MAS occurred in a preterm male infant born at 27 weeks of gestation who presented with refractory systemic hypertension^2^. Published series of children with renovascular hypertension report a variable incidence of MAS: 2% patients in Turkey^8^, 12% in the United States^9^, 47% in Canada^10^, 18% in Australia^11^, and 20% in India^12^. These data highlight differences in the referral populations, as most reports originate from specialist single centers. Cases of MAS have also been reported in the Middle East, North Africa, South and Far-East Asia, and Central and South America^8, 13–16^. It remains difficult to ascertain the true burden of MAS owing to the paucity of population-based national and international registries.

A Vascular Low-Frequency Disease Consortium project on pediatric MAS is currently in progress in the US and will hopefully provide important information on the incidence, prevalence, up-to-date management, and outcomes of this disease^16^.

## Aetiology

The aetiology of MAS in the literature has been defined as idiopathic, congenital, or acquired. The latter includes both inflammatory and genetic factors. Recent studies have increasingly identified previously diagnosed idiopathic or even acquired causes of MAS to be the result of underlying genetic abnormalities^1, 5, 17–21^.

A proposed embryological mechanism for congenital MAS has been attributed to events occurring around day 25 of fetal development. At this time, the two embryonic dorsal aortas fuse and lose their intervening walls to form a single vessel. Overfusion of the two dorsal aortas—or their failure to fuse with subsequent obliteration of one of these vessels—may result in aortic narrowing^18^. This theory regarding developmental overfusion of the two primitive dorsal aortas is supported by the presence of a single origin of the lumbar arteries in some patients with decreased aortic diameters^22^. Multiple renal arteries supplying one or both kidneys in nearly half of patients exhibiting supra-renal and infra-renal abdominal aortic coarctations exceeds the 25–35% observed in the general population. These observations also support a developmental cause for the narrowing observed in MAS^23^. Interestingly, several reports of MAS presenting in the neonatal period highlight concomitant premature closure of the ductus arteriosus, although there are few data to propose a reduced-flow hypothesis for the development of some cases of early symptomatic MAS^24, 25^.

Viral-mediated events may impede the transition of fetal mesenchymal tissue to vascular smooth muscle or alter its organization and growth in utero and may be a cause of developmental aortic narrowing. Certain viruses, including rubella, are cytocidal and inhibit cell replication, with intimal fibroplasia and aortic hypoplasia occurring as well-recognized sequelae. Fibro-proliferative intimal disorders have been documented in the aorta, and large elastic arteries are present in 16.5% of patients with congenital rubella syndrome^26, 27^.

MAS can be associated with genetic disorders including neurofibromatosis-1 (NF-1), Williams syndrome, and Alagille syndrome. Patients with NF-1 exhibit an unusually high frequency of arterial abnormalities, including aortic coarctation and arterial stenosis involving the renal, cerebral, visceral, and coronary vessels. Because of the protean nature of NF-1 and infrequent genetic analyses of patients with abdominal aortic coarctation, the incidence of MAS among these individuals is unknown. The stenosis in NF-1 may be attributable to enlarging neurofibromas in large and medium calibre arteries or spindle cell proliferation in the arterial wall of the small vessels 17. Additionally, Warejko et al. reported a high proportion of likely causal mutations in vasculopathy genes in syndromic and nonsyndromic cases of MAS^28^.

MAS can also be associated with inflammatory disorders such as Takayasu’s arteritis. Pan-aortitis with adventitial or periadventitial fibrosis and associated inflammatory cell infiltrates, suggesting active or chronic aortitis, is a recognized cause of abdominal aortic coarctation. The proposition that most abdominal aortic coarctations are a variant of inflammatory aortitis, such as that seen in Takayasu’s arteritis, remains controversial and is not supported by histological findings. This cause of aortic narrowing is more common in Asian and South American populations^29, 30^.

## Classification

**Figure 1.**
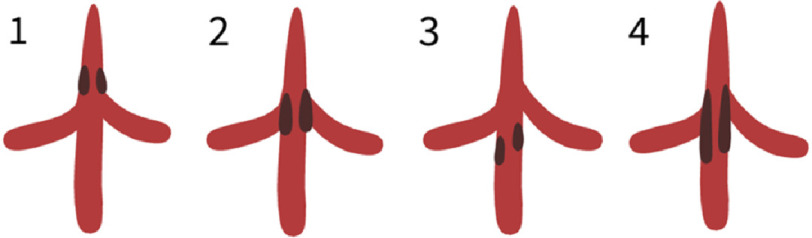
Classification of MAS. (1) Suprarenal; (2) inter-renal; (3) infra-renal; (4) supra- to infra-renal.

MAS is most commonly classified anatomically, based on the site of narrowing of the most cephalad aortic segment. Possible sites of aortic coarctations and collateral pathways are presented in Figure 1.

The abdominal aorta is involved in nearly 97% of MAS cases, with the remainder of cases involving the distal thoracic aorta. Multiple anatomical and morphological classifications of MAS have been proposed (for example, Hallett’s, Robicsek’s, Graham’s and Mickley and Fleiter’s) based on the relation of aortic narrowing to the renal arteries, renal involvement, and type of stenosis^17^. The most common anatomic site of aortic narrowing is suprarenal (29%–60%) with infra- and juxtarenal stenosis being less common (8%–15%)^1, 2^.

Genetic disorders are often associated with suprarenal aortic stenosis and a high incidence (70–85%) of extra-aortic vessel involvement, typically involving the renal and superior mesenteric arteries and the coeliac axis^1^.

The renal arteries are commonly affected in cases of MAS, with an incidence of approximately 70%. Of cases with renal artery involvement, approximately 60% have bilateral disease^1^. The mesenteric vessels are the second most commonly affected vessels in MAS. The superior mesenteric artery is affected in approximately 30% of cases, and the coeliac trunk is involved in 22% of cases with extra-aortic involvement. The inferior mesenteric artery is rarely involved. Iliac, carotid, cerebral and brachial artery involvement have also been described in the literature^1^.

### Clinical presentation

Hypertension remains the most commonly reported clinical presentation of MAS regardless of the geographical area and mean age of patients within various case series (Table 1). Refractory hypertension can manifest as stroke, hypertensive encephalopathy, and congestive heart failure. Refractory hypertension secondary to MAS was previously associated with a 45–55% mortality rate before effective antihypertensive therapies were readily available^5^. Despite improvements in mortality, a high degree of renal vessel involvement is associated with progression to end-stage kidney disease in patients with severe lesions^2^.

**Table 1:** Most patients with MAS present with hypertension, whereas the second and third most common clinical features vary across case series. The mean age of the series does not necessarily represent the mean age at presentation/diagnosis of MAS [6,10,18,19,31–34].

Author, year (Ref)	Country	Mean age	Most common clinical feature		
			1st	2nd	3rd
Hahn 1998 (31)	South Africa	8.4	Hypertension	Cardiac failure	Bruits
Sethna 2008 (6)	USA	14.3	Hypertension	Claudication	Renal failure
Tummolo 2009 (19)	UK	4	Hypertension	Cardiac failure	Hypertensive encephalopathy
Taketani 2005 (32)	Japan	32.6	Hypertension	claudication, fatigability, headache	Syncope
Stanley 2008 (18)	USA	11.9	Hypertension	Claudication	Intestinal angina
Shroff 2006 (33)	UK	8.4	Hypertension	Cardiac failure, palpitations, murmur	Headache
D’Souza 1997 (10)	Canada	8.5	Hypertension	Claudication	Headache, respiratory arrest
Tyagi 1999 (34)	India	9.78	Hypertension	Cardiac failure	Claudication

Other symptoms of MAS may include claudication, dyspnea, headache, failure to thrive, nausea and/or vomiting, abdominal angina, leg weakness, or intermittent claudication-like symptoms^1, 6, 19, 31, 34, 35^. In some cases, the patient’s history may include fevers, fatigue and joint pain suggestive of an inflammatory pathology such as Takayasu’s arteritis^36^.

In patients with MAS, further examination following the detection of raised blood pressure for age may reveal absent or diminished femoral pulses, systolic murmur, abdominal bruit, and reduced ankle-brachial pressure index^1, 2, 17, 21, 37^. In addition, enlarged collaterals may be palpable with audible bruits present^17^. In patients with associated diseases such as NF-1, additional specific signs may be present on examination, such as café au lait spots^2^.

Premature mortality remains high in patients who present in the neonatal period with hypertension refractory to medical therapy, as their small size makes endovascular procedures challenging and prohibits surgical management^21, 24, 38, 39^. In older children, however, morbidity and mortality rates are reported to be improving. However, care must be taken when interpreting any results as published series consist of a heterogeneous collection of patients with renovascular hypertension and patients with MAS undergoing different invasive and non-invasive treatments at different treatment thresholds and with different techniques. Even series focusing exclusively on MAS are difficult to compare as some include patients over several decades with different associated features (e.g., genetic syndromes) and different management strategies which may affect outcomes. For example, Table 2 shows a mortality rate of 12% in the series presented by Taketani et al., which includes patients between 1960 and 2004, whereas there is no reported mortality in any of the reported (albeit smaller) series including patients after 1987^20, 32, 34, 39–42^.

**Table 2:** Mortality rates and reported improvement of hypertension following intervention for middle aortic syndrome by chronological order of the included case series [5,18–20,32,34,39–44].

Author, year (reference)	Case year	Aetiology when known	Mortality	Hypertension improved following intervention
Taketani 2005 (32)	1960-2004	Takayasu’s arteritis	4/33	14/33
Stanley 2008 (18)	1963-2008	^*^NF-1 14; Williams Syndrome 1; Alagille syndrome 1	6/53	46/53
Sumboonanonda 1992 (19)	1975-1988	Williams Syndrome 3	1/8	1/2
Tummolo 2009 (19)	1976-2008	NF-1 7; Williams Syndrome 3; Hypomelanosis of Ito 1; Feuerstein-Mims syndrome 1; Chromosome 10 abnormality 1	3/36	30/36
Porras 2013 (5)	1981-2012	Williams Syndrome 23%; Takayasu’s arteritis 15%; NF-1 9%; Alagille syndrome 7%; Moya-Moya disease 4%	3/53	9/35
Tyagi 1999 (34)	1987-1999	Unknown	0/38	35/38
Siwik 2003 (20)	1989-2000	NF-1 2; Williams syndrome 2; Takayasu’s arteritis 1; Congenital rubella 1	0/9	^ˆ^NR
Saxena 2000 (41)	Published 2000	Unknown	0/17	14/17
Soumer 2015 (44)	2012-2013	Unknown	0/3	3/3
Hetzer 2013 (39)	2013	Unknown	0/14	12/14
Kim 2018 (42)	2018	NF-1	0/5	4/5

**Notes.**

* NF-1, Neurofibromatosis-1.

ˆ NR, not reported.

On the other hand, not much is known about the impact of the aetiology of MAS on subsequent outcomes. Regardless, most studies report improvement or resolution of hypertension and symptoms of claudication following intervention, as shown in Table 2^5, 20, 32, 34, 39–42^. Saxena et al. reported improved left ventricular function in 8 of 11 patients with previously significant left ventricular dysfunction, Tyagi et al. reported improvement in severe congestive heart failure in 21 of 22 patients and Porras et al. reported resolution of left ventricular dysfunction in all 5 patients^5, 34, 41^. On the other hand, Porras et al. reported kidney dysfunction at presentation in 27% patients and the presence of chronic kidney disease in 23% of patients at follow up^5^.

### Investigations

Hypertension in children is often an incidental finding and is defined as systolic and/or diastolic blood pressure persistently equal to, or greater than, the 95th percentile for sex, age and height measured on at least three separate occasions^7, 44^. The 2016 ESH Clinical Practice Guidelines recommend that all children diagnosed with hypertension should undergo routine biochemical and radiological investigations including ultrasound of the urinary tract^7^. For patients in whom secondary hypertension is suspected, especially in the context of markedly elevated blood pressure or secondary complications, more specific testing is indicated. Investigations that may be indicated include peripheral plasma renin and aldosterone to evaluate for primary hyperaldosteronism; urine and plasma catecholamines and/or metanephrines to evaluate for phaeochromocytoma; urinary free cortisol to evaluate for Cushing’s syndrome; thyroid function tests to evaluate for thyrotoxicosis; plasma deoxycorticosterone and corticosterone to evaluate for congenital adrenal hyperplasia; urine toxicology screening for amphetamine or ecstasy use; and genetic studies to evaluate for monogenic causes of hypertension in specific clinical situations^7^.

Of the above investigations, renal ultrasound has the greatest potential to capture MAS as the cause of hypertension. Ultrasound is an ideal first-line imaging tool because it is safe without using ionising radiation, readily available, and does not require a general anesthetic. On ultrasound, the aortic stenosis may be directly visualised in thin and compliant patients. In addition, typical parvus and tardus waveforms and elevated peak systolic flow may be observed, particularly in the main renal arteries. If ultrasound is equivocal or if there is a high index of clinical suspicion despite a ‘normal’ ultrasound, CTA or MRA provides sensitivity and specificity exceeding 80% for diagnosing MAS and concomitant renal, mesenteric, and iliac vessel involvement^45^.

Catheter angiography remains the gold standard for diagnosis. However, it is now mainly used as a precursor in patients undergoing simultaneous endovascular intervention. The scope of catheter angiography has recently expanded with the more routine use of DynaCT, intravascular imaging (intravascular ultrasound, optical coherence tomography), and perfusion assessment (fractional flow reserve and quantitative digital subtraction angiography). The definite value-added benefits of these additional modalities have not yet been established. However, they provide exciting potential scope for more functional assessment beyond the 2-dimensional angiographic image^2, 17, 19, 36, 46, 47^.

Once MAS is diagnosed, additional laboratory investigations to screen for active Takayasu’s arteritis include erythrocyte sedimentation rate (ESR), C-reactive protein (CRP), immunoglobulins, antinuclear antibodies and eosinophil levels should be performed^2, 21, 36, 48^. Given the relatively common presence of genetic disorders in patients with MAS (15.4% in the systematic review by Rumman et al.), genetic testing for NF, William’s syndrome and Alagille syndrome may be indicated depending on the phenotype^1^.

**Figure 2.**
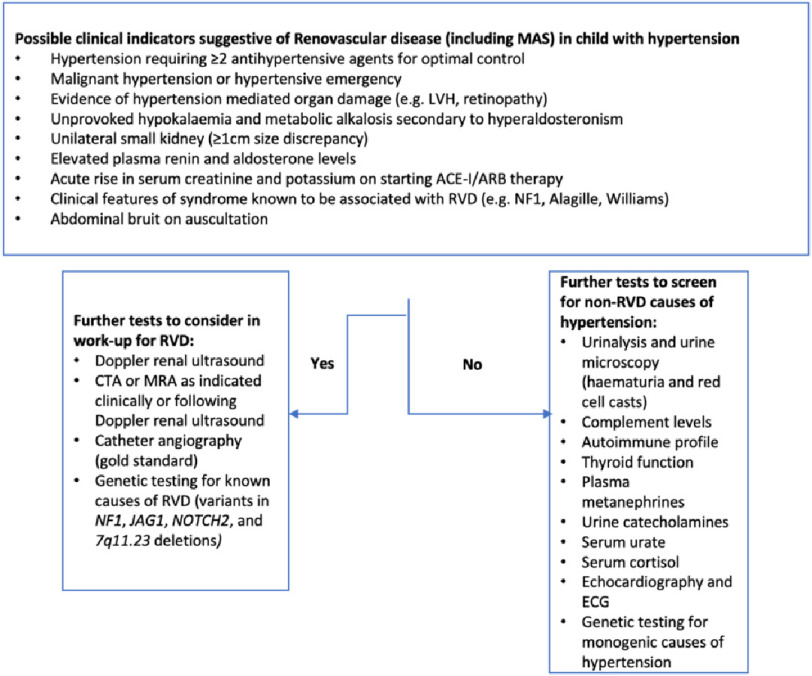
Indicators of renovascular disease (including MAS) in child with hypertension and suggested investigation pathway.

Investigations for end-organ damage related to hypertension include kidney function tests and urinary protein quantification to evaluate for acute or chronic kidney impairment^2, 5, 17, 21^, echocardiography to evaluate left ventricular function and assess for ventricular hypertrophy^1, 2, 5, 49^, and ophthalmoscopy to evaluate for hypertensive retinopathy^5^. Investigations that are part of the diagnostic process in MAS are summarised in Figure 2.

### Management of MAS

#### Medical management

In general, the choice of antihypertensive medication depends upon the clinical presentation, available medications, and experience of the treating physicians with different classes of antihypertensive agents. As patients with MAS often present with markedly elevated BP or hypertensive urgency, initial management requires a combination of antihypertensives, which may necessitate the need for intravenous medications. The use of medications may also be guided by the unilateral or bilateral nature of the associated renal artery stenosis (RAS) seen frequently and by the presence of associated complications, particularly cardiac or kidney dysfunction.

In a recent review by Rumman et al., patients receiving medical management became normotensive in 14.4% of cases and improved in 35.8% of cases^1^. However, 43.6% of cases remained refractory to treatment. A combination of antihypertensive medications is frequently required to optimize BP control, with no clear advantage of any one class of antihypertensive medication over another.

Often, a combination of agents is required to manage BP, including calcium channel blockers, beta blockers, and alpha blockers. Hydralazine, minoxidil, and angiotensin-converting enzyme inhibitors/angiotensin II receptor blockers (ACEi/ARB) may be used with specific considerations, as discussed below. The use of diuretics needs particular consideration, as they may often be avoided in the acute phase, as severe hypertension is frequently associated with biochemical abnormalities and/or dehydration.

Although ACEi/ARBs are very effective in renovascular disease, expert guidance and caution are needed to avoid renal ischemia as a result of impairment of the normal autoregulation of constriction within the post-glomerular (efferent) arteriole. Reduced angiotensin II production results in a drop in glomerular perfusion, predisposing patients to ischemic nephropathy and kidney impairment. Kidney function and serum potassium levels should be monitored closely when commencing ACEi/ARB therapy, and they may need to be discontinued if there is a rapid and sustained rise in serum creatinine values and/or refractory hyperkalemia that does not respond to dietary modification or medical management.

Patients with idiopathic MAS have higher rates of refractory hypertension despite medical management compared with MAS owing to other aetiologies^1^. Medical management is often only successful in cases with mild-to-moderate stenosis and more severe disease often requires surgical intervention^5, 21^.

In patients with active aortoarteritis, efforts should be made to control the acute phase of the disease before surgical reconstruction. Corticosteroids and other immunosuppressive agents have also been used effectively. The response to therapy and the level of inflammatory activity are guided by the determination of acute-phase reactants such as CRP and ESR^17^.

### Interventions for MAS

Invasive management of MAS includes endovascular and open surgical options^5^. At present there is no consensus regarding indications for invasive management of MAS. The decision to proceed to invasive management is most often indicated by poor blood pressure control despite optimal medical management^2, 17, 39, 49^ or significant/intolerable side effects of medical therapy^5^. Other indications for treatment include evidence of hypertension-mediated end-organ damage, including left ventricular hypertrophy and hypertensive retinopathy^2, 5, 17, 49^, kidney dysfunction, abdominal angina, and leg claudication^5^. In the case series by Porras et al., all patients with mid-aortic stenosis of ≥60% underwent invasive management for middle aortic syndrome, whereas all patients with MAS <40% were managed conservatively^5^.

The aim of intervention is to optimise blood pressure control and reverse end-organ damage. Open surgical treatment is the mainstay of intervention and the timing of surgery is determined by the severity of hypertension, degree of related physical incapacitation and the potential for further growth^17^. Open surgical treatment is generally preferred when full adult growth has occurred. The main role of endovascular interventions in MAS is to supplement medical therapy until a definitive surgical intervention can be performed. Surgery should be deferred until full growth potential is reached. In cases of severe refractory hypertension, early surgical intervention can be offered.

### (i) Endovascular

While the gold standard for the treatment of MAS remains open surgical repair, percutaneous interventional techniques permit critical aortic, renal, and mesenteric arterial stenoses to be treated in a minimally invasive manner, even in very young children. Endovascular management of MAS consists of percutaneous transluminal angioplasty (PTA). Cutting balloon angioplasty has been described for use in resistant stenoses and re-stenotic lesions^5, 48^. Stent insertion is reserved as a salvage manoeuvre when an intra-procedural complication has been encountered, such as flow-limiting dissection or arterial thrombosis^20^.

Lesions deemed most suitable for endovascular treatment are short and isolated^49^. Guidelines for aortic coarctation recommend using a balloon diameter of 2–5 times the stenosis diameter, but limit oversizing compared to the adjacent segment of ‘normal’ aorta^20^.

The reported effectiveness of endovascular interventions varies between studies. Most patients require ongoing medical treatment. In addition, repeated interventions are likely to be required^1, 5, 20, 47^. In a case series by Porras et al., freedom from re-intervention was 58% at 1-year and 33% at 5-years^5^.

The success rate of PTA in children with RAS varies from 25–94%. Shroff et al. reported a 55% overall improvement in BP control in 33 children with RAS, but the percentage of BP improvement in the subgroup (RAS patients with aortic stenosis) was much lower than that with isolated RAS (33% vs. 85%). The extensive nature of aortic and renal lesions in MAS generally decreases the PTA success rate compared to that in patients with isolated RAS^33^. In a series by Saxena et al., 17 patients underwent endovascular management of MAS with no mortality and improvement in BP in 14 patients^41^. Porras et al. reported 30 patients who underwent endovascular management of MAS, of which 1 did not require antihypertensive medication, 13 had good BP control with medication, and 9 required further surgery for BP control^5^. In a case series by Tummolo et al., 13 patients underwent endovascular management, 1 was able to discontinue medication, and 9 had good BP control on antihypertensive medication^19^. In the series by Tyagi et al., 11 out of 38 patients did not require antihypertensive medication following endovascular management, and 24 had good BP control after intervention with the help of medication^34^. In the systematic review by Rumman et al., following endovascular procedure, 36.4% of patients had their blood pressure controlled with medication, 17.9% without medication, and 13.3% had uncontrolled blood pressure^1^. It is important to note that dependence on antihypertensive medications and the number of medications needed to control blood pressure following an interventional procedure might change over time as the child grows^50^.

Complications following endovascular management include arterial dissection^5, 20^, thrombosis, and aneurysm formation^5, 17, 20^. Patients with NF-1 appear to be at a higher risk of vascular complications from catheter-based interventions, as well as aneurysm formation. Additionally, when stents are used, they have the potential to migrate or thrombose^5, 20^.

Owing to the modest clinical response and need for re-interventions, endovascular intervention is generally indicated in the acute setting to reduce blood pressure or restore end-organ perfusion and to allow definitive surgical treatment to be deferred until the child is physically fully grown.

### (ii) Surgical

Surgical repair in a patient with MAS is determined primarily by the severity of hypertension, the degree of related physical incapacitation, and the potential for further growth^17^. The timing of surgery depends primarily on the severity of (uncontrolled) hypertension and the age of the patient. As a general rule, surgery should be deferred until full growth has been achieved however in cases of severe refractory hypertension and end organ damage early intervention is advised^1, 5, 17^. From 15 to 55% of patients who require invasive intervention undergo open surgery, with the wide range possibly reflecting regional differences in expertise as well as anatomical substrate (stenosis site and length, involvement of other vessels)^1, 5, 51^.

Surgical interventions include aorto-aortic bypass, patch aortoplasty, and primary aortic repair after aortic lengthening. In a review of 630 cases of MAS, 55% of patients who underwent surgical intervention had aorto-aortic bypass (42%), followed by reconstruction patch graft (23%), and renal autotransplantation (11%)^1, 2^. Patch aortoplasty has been suggested to be the optimal intervention for infants because of their anticipated growth^18^.

### Technical details of surgical intervention for MAS

#### (a) Aortoaortic bypass

MAS, particularly in the presence of diffuse and lengthy stenoses, is best treated by the construction of an aortoaortic bypass from above to below the stenosis. The level of aortic anastomosis depends on the exact anatomical distribution of the MAS. The aorta can be approached by either a midline abdominal incision or a left thoracoabdominal approach. The latter is indicated if aortic coarctation reaches the diaphragm or extends proximal to it, and this is guided by the extent of the disease and the length of bypass being performed.

#### (b) Patch aortoplasty

Patch aortoplasty of the stenotic aortic segment may be considered in moderate and less lengthy stenoses using synthetic material or bovine pericardium^17^. The aortic patch may be fashioned to extend into the stenotic visceral or renal arteries, and may be used as a site for reimplantation of the mesenteric arteries or for renal artery bypass.

#### (c) Renal artery reconstruction

Patients with MAS may also require renal and splanchnic arterial reconstruction). In a systematic review by Rumman et al., renal artery reimplantation was required in 9.7% and renal autotransplantation in 11.2% of patients undergoing surgical treatment^1^. Stanley et al. favored the use of iliac artery autografts instead of vein grafts in renal reconstructions, as they are less prone to dilatation over time 18. Visceral artery reconstruction is of uncertain value in asymptomatic patients, whereas patch angioplasty or aorto-visceral bypass may be considered in symptomatic patients^1, 5, 17, 18^. Renal artery reconstruction may be performed with aortorenal bypass, splenorenal anastomosis, hepatorenal bypass, or autotransplantation of the kidney into the pelvis after aortic reconstruction. Autogenous vein (LSV) or internal iliac artery grafts are indicated for renal artery bypass, especially in the youngest patients^17^. Prosthetic renal and visceral bypasses with Dacron or polytetrafluoroethylene (PTFE) grafts have also been used successfully.

### Surgical outcomes

Surgical intervention carries a complication rate of 9% and a mortality rate of 2.9%–4%. Complications include graft stenosis, bleeding, thrombosis, and iatrogenic tears. Notably, cases associated with arteritis had the highest complication rate during surgical intervention 1. Freedom from re-intervention after surgery is reported to be 72% at 10 years^5^. Importantly, in complex conditions such as MAS, surgery is often not associated with the need to stop antihypertensive medication but often results in improving the medical management of hypertension in children. All three patients in the series by Soumer et al. still required medication to control BP post-operatively^43^.

In the series by Kim et al., which included five patients, three had their BP controlled without medication after surgery, and two still required antihypertensive medication^42^. The series by Stanley et al. which included 53 patients, showed that hypertension was controlled in 46 patients post-operatively^18^. In Porras’ series, 14 patients in total underwent surgical repair on MAS and of those, blood pressure was controlled in three without, and six with medication^5^.

In the series by Tummolo et al., 6 of 10 patients did not require antihypertensive medication after surgery^19^. Connolly et al. reported that all eight patients in their series who underwent surgery for MAS achieved long-term correction of their hypertension^21^. In a systematic review by Rumman et al., post-surgical blood pressure was controlled with antihypertensive medication in 24.7% of cases, without medication in 31.9% of cases, and remained uncontrolled in 4.6% of cases^1^.

In patients with Takayasu’s arteritis, surgical management should only be attempted after the disease has burnt out^21^. In anatomically suitable defects, patch aortoplasty can be used as an interim measure in young patients with severe hypertension, before definitive revascularization is attempted later on when the patient has physically grown^1, 5, 17, 18, 32^. In the case series by Stanley et al., Dacron was used more commonly in earlier experience, whereas Teflon grafts predominated in recent experience. They used 8-12 mm grafts in young children, 12-16 mm grafts for early adolescents, and 14-20 mm grafts in late adolescents and adults to accommodate growth^18^.

Surgical management was complicated in 7.1%, failed in 14.3%, and resulted in death in 2.9% cases^1^. This is in contrast to a report by Taketani et al., which showed a mortality rate of 12.1% following surgical repairs before 1968^32^. Although Stanley et al. reported a mortality rate of 11% at follow-up, there were no perioperative deaths^18^. Complications following surgery in patients with middle aortic syndrome include bleeding and graft thrombosis^5^. Late reoperation may be required because of the expected outgrowth of the original graft, inadequately sized patch, and aneurysmal dilatation of the aorta at the site of aortoplasty or anastomosis^18, 32^. According to a case series by Porras et al., freedom from reintervention after a surgical procedure was 83% at 1 year and 72% at 10 years^5^.

Newer surgical techniques in the management of middle aortic syndrome include primary aortic repair after aortic lengthening^5^ and single-stage aortic bypass using the meandering mesenteric artery (‘mesenteric artery growth improves circulation’)^52^.

### Challenges and future directions

The wide range of reported outcomes and paucity of information regarding factors predictive of outcomes are related to the nature of published studies. It is therefore difficult to develop clear guidelines for the diagnosis and management of MAS in children as most of the data guiding management are derived from retrospective or prospective observational studies on relatively small patient numbers over long time periods with heterogeneity of reported cohorts in terms of aetiology, response to medications, and vascular involvement as described below.

First, the lack of multicenter studies makes it difficult to ascertain the true worldwide burden of MAS. Additionally, many studies including larger numbers of patients report pooled outcomes for patients with renovascular hypertension without a clear comparison between patients with MAS of different aetiology and other forms of renovascular hypertension, or without a comparison between conservatively and surgically/endovascularly managed patients with MAS. Due to the low number of cases, any difference in prognosis on the basis of anatomical or histopathological features remains speculative. Furthermore, techniques for invasive management of MAS have developed over time so that a proportion of outcome data are historical in nature and may display an era effect.

Although MAS has been associated with significant premature mortality in the past (with a reported mean survival age of 34 years in one historical systematic review of cases), the hope is that modern surgical and endovascular management options will lead to lower morbidity and mortality^17^. This seems to be the case in the medium-term period (5.2% mortality at 4 years of follow-up in a systematic review of cases with a mean age of 9.1); however, longer follow-up times are needed to assess the impact of earlier intervention on long-term survival^1^.

Future directions of investigation should include early consideration for genetic testing to help guide an understanding of the underlying aetiology and other systemic manifestations that could be expected. Warejko et al. showed the presence of a genetic variant in one of the 38 known vasculopathy genes in 42.9% of families with MAS, although further studies are needed to characterize the genotype-phenotype correlation^28^. In another cohort of 37 children with renovascular hypertension, 20 (54%) of whom had MAS, whole exome sequencing identified five patients (14%) to have pathogenic variants in genes known to be associated with renovascular disease (NF-1, ELN, and a chromosome 7q11.23 deletion). Two other patients (5%) were found to have likely pathogenic variants in genes putatively associated with renovascular disease (SMAD6 and GLA)^53^.

Since its founding in 2015, a European/International Fibromuscular Dysplasia Registry has enrolled patients from more than 20 countries and has allowed characterisation of patient profiles, including those complicated by widespread disease, aneurysms and dissections, which has led to targeted screening, management and follow up of patients with fibromuscular dysplasia^54^. A similar database of patients with MAS would be helpful in improving our understanding of MAS aetiology, presentation, and outcomes based on clinical characteristics and management. This is particularly important in view of the low-volume and high-complexity procedures used to treat MAS.

## Summary

MAS is a rare but important cause of hypertension in children and young people that is associated with stenosis of the renal or visceral arteries and is associated with certain genetic syndromes. When aortic narrowing exceeds 60%, conservative management is unlikely to be successful and invasive therapy is indicated. In younger children, endovascular therapy may be attempted in the first instance to acutely reduce the pressure gradient across the narrowing; however, a sustained benefit is rare. Once the child has grown to accommodate a graft of adequate size, surgical therapy is indicated for patients in whom medical and/or endovascular management has not resulted in adequate blood pressure control, as well as for those who have significant side effects from medical therapy and/or suffer from end-organ damage due to longstanding uncontrolled hypertension. A multidisciplinary approach and regular follow-up are crucial to monitor for recurrent hypertension and evidence of end-organ damage.

References1.
RummanRK
NickelC
Matsuda-AbediniM
LorenzoAJ
LangloisV
RadhakrishnanS
AmaralJ
MertensL
ParekhRS
2015Disease beyond the Arch: a systematic review of middle aortic syndrome in childhoodAmerican Journal of Hypertension287833846doi: 10.1093/ajh/hpu296256313832.
FormanN
SinskeyJ
ShalabiA
2020A review of middle aortic syndromes in pediatric patientsJournal of Cardiothoracic and Vascular Anesthesia34410421050doi: 10.1053/j.jvca.2019.07.130314129803.
SenPK
KinareSG
EngineerSD
ParulkarGB
1963The middle aortic syndromeHeart255610618doi: 10.1136/hrt.25.5.610PMC1018042140630084.
KumarS
BuryRW
RobertsDH
2002Unusual case of refractory hypertension: late presentation of the mid-aortic syndromeHeart (British Cardiac Society)872doi: 10.1136/heart.87.2.e3PMC1767006117965665.
PorrasD
SteinDR
FergusonMA
ChaudryG
AlomariA
VakiliK
FishmanSJ
LockJE
KimHB
2013Midaortic syndrome: 30 years of experience with medical, endovascular and surgical managementPediatric Nephrology281020232033doi: 10.1007/s00467-013-2514-823775038PMC38223376.
SethnaCB
KaplanBS
CahillAM
VelazquezOC
MeyersKEC
2008Idiopathic mid-aortic syndrome in childrenPediatric Nephrology23711351142doi: 10.1007/s00467-008-0767-4183202357.
LurbeE
Agabiti-RoseiE
CruickshankJK
DominiczakA
ErdineS
HirthA
InvittiC
LitwinM
ManciaG
PallD
RascherW
RedonJ
SchaeferF
SeemanT
SinhaM
StabouliS
WebbNJ
WühlE
ZanchettiA
2016 European Society of Hypertension guidelines for the management of high blood pressure in children and adolescentsJournal of Hypertension341018871920doi: 10.1097/HJH.0000000000001039274677688.
BayazitAK
YalcinkayaF
CakarN
DuzovaA
BircanZ
BakkalogluA
CanpolatN
KaraN
SirinA
EkimM
OnerA
AkmanS
MirS
BaskinE
PoyrazogluHM
NoyanA
AkilI
BakkalogluS
SoyluA
2007Reno-vascular hypertension in childhood: a nationwide surveyPediatric Nephrology22913271133doi: 10.1007/s00467-007-0520-4175346669.
PiercyKT
HundleyJC
StaffordJM

2005Renovascular disease in children and adolescentsJournal of Vascular Surgery416973982doi: 10.1016/j.jvs.2005.03.0071594459610.
D’SouzaSJ
TsaiWS
SilverMM
ChaitP
BensonLN
SilvermanE
HebértD
BalfeJW
1998Diagnosis and management of stenotic aorto-arteriopathy in childhoodThe Journal of Pediatrics132610161022doi: 10.1016/S0022-3476(98)70401-9962759611.
McTaggartSJ
GulatiS
WalkerRG
PowellHR
JonesCL
2000Evaluation and long-term outcome of pediatric renovascular hypertensionPediatric Nephrology1410–1110221029doi: 10.1007/s0046700500661097532012.
KanitkarM
2005Renovascular hypertensionIndian Pediatrics42147541569585813.
AhnKJ
YoonJK
KimGB
KwonBS
BaeEJ
NohC
2016Idiopathic midaortic syndrome with malignan hypertension in 3-year-old boyKorean Journal of Pediatrics592016S84S87doi: 10.3345/kjp.2016.59.11.S8428018454PMC517772114.
VasconcelosVT de
CalRGR
GomesAL
AguiarS
CamargoMFC de
Baptista-SilvaJCC
2015Long-segment thoracoabdominal aortic coarctation in a child with Down syndromeJournal of Vascular Surgery Cases12171173doi: 10.1016/j.jvsc.2015.04.01131724602PMC684992115.
MarinaL
AnaT
MaríaJC
LuisEA
2019Severe infantile coarctation and mid aortic stenosis in williams syndromeJournal of Cardiology and Cardiovascular Medicine42080082doi: 10.29328/journal.jccm.100104416.
LawrencePF
BarilDT
WooK
2020Investigating uncommon vascular diseases using the vascular low frequency disease consortiumJournal of Vascular Surgery72310051010doi: 10.1016/j.jvs.2019.11.02931964572PMC736770417.
DelisKT
GloviczkiP
2005Middle aortic syndrome: from presentation to contemporary open surgical and endovascular treatmentPerspectives in Vascular Surgery and Endovascular Therapy173187203doi: 10.1177/1531003505017003021627315418.
StanleyJC
CriadoE
EliasonJL
UpchurchGR
BerguerR
RectenwaldJE
2008Abdominal aortic coarctation: surgical treatment of 53 patients with a thoracoabdominal bypass, patch aortoplasty, or interposition aortoaortic graftJournal of Vascular Surgery48510731082doi: 10.1016/j.jvs.2008.05.0781869235219.
TummoloA
MarksSD
StadermannM
RoebuckDJ
McLarenCA
HamiltonG
DillonMJ
TullusK
2009Mid-aortic syndrome: long-term outcome of 36 childrenPediatric Nephrology241122252232doi: 10.1007/s00467-009-1242-61960319420.
SiwikES
PerrySB
LockJE
2003Endovascular stent implantation in patients with stenotic aortoarteriopathies: early and medium - Term resultsCatheterization and Cardiovascular Interventions593380386doi: 10.1002/ccd.105461282216521.
ConnollyJE
WilsonSE
LawrencePL
FujitaniRM
Middle aortic syndrome: distal thoracic and abdominal coarctation, a disorder with multiple etiologies10.1016/s1072-7515(02)01144-41208106822.
ArnotRS
LouwJH
1973The anatomy of the posterior wall of the abdominal aorta. Its significance with regard to hypoplasia of the distal aortaSouth African Medical Journal = Suid-Afrikaanse Tydskrif Vir Geneeskunde4721899902471100323.
StanleyJC
GrahamLM
Whitehouse JrWM
ZelenockGB
ErlandsonEE
CronenwettJL
LindenauerSM
1981Developmental occlusive disease of the abdominal aorta and the splanchnic and renal arteriesThe American Journal of Surgery1422190196doi: 10.1016/0002-9610(81)90273-7725852624.
IzraelitA
KimM
RatnerV
LevasseurSM
SeigleR
KrishnamurthyG
2012Mid-aortic syndrome in two preterm infantsJournal of Perinatology325390392doi: 10.1038/jp.2011.1302253837825.
ZeltserI
ParnessIA
KoH
HolzmanIR
KamenirSA
2003Midaortic syndrome in the fetus and premature newborn: a new etiology of nonimmune hydrops fetalis and reversible fetal cardiomyopathyPediatrics111614371442doi: 10.1542/peds.111.6.14371277756826.
RosenbergHS
OppenheimerEH
EsterlyJR
1981Congenital rubella syndrome: the late effects and their relation to early lesionsPerspectives in Pediatric Pathology6183202732282727.
LimbacherJP
HillME
JanickiPC
1979Hypoplasia of the abdominal aorta associated with rubella syndromeSouthern Medical Journal725617618doi: 10.1097/00007611-197905000-0003244177828.
WarejkoJK
SchuelerM
VivanteA
TanW
DagaA
LawsonJA
BraunDA
ShrilS
AmannK
SomersMJG
RodigNM
BaumMA
DaoukG
TraumAZ
KimHB
VakiliK
PorrasD
LockJ
RivkinMJ
ChaudryG
SmootLB
SinghMN
SmithER
ManeSM
LiftonRP
SteinDR
FergusonMA
HildebrandtF
2018Whole exome sequencing reveals a monogenic cause of disease in ∼43% of 35 families with Midaortic syndromeHypertension714691699doi: 10.1161/HYPERTENSIONAHA.117.1029629483232PMC584355029.
WadaJ
KazuiT
1978Long-term results of thoracoabdominal bypass graft for atypical coarctation of the aortaWorld Journal of Surgery26891896doi: 10.1007/BF0155655072649830.
LandeA
1976Takayasu’s arteritis and congenital coarctation of the descending thoracic and abdominal aorta: a critical reviewAmerican Journal of Roentgenology1272227233doi: 10.2214/ajr.127.2.227795731.
HahnD
ThomsonPD
KalaU
BealePG
LevinSE
1998A review of Takayasu’s arteritis in children in Gauteng, South AfricaPediatric Nephrology128668675doi: 10.1007/s004670050526981139332.
TaketaniT
MiyataT
MorotaT
TakamotoS
2005Surgical treatment of atypical aortic coarctation complicating Takayasu’s arteritis—Experience with 33 cases over 44 yearsJournal of Vascular Surgery414597601doi: 10.1016/j.jvs.2005.01.0221587492233.
ShroffR
RoebuckDJ
GordonI

2006Angioplasty for renovascular hypertension in children: 20-Year experiencePediatrics1181268275doi: 10.1542/peds.2005-26421681857434.
TyagiS
KhanAA
KaulUA
AroraR
1999Percutaneous transluminal angioplasty for stenosis of the aorta due to aortic arteritis in childrenPediatric Cardiology206404410doi: 10.1007/s0024699005011055638635.
DayE
StojanovicJ
KarunanthyN
QureshiS
ReidyJ
SinhaMD
2015Middle aortic syndrome—an 8-year story of pills, pretty balloons and strutsPediatric Nephrology30813611365doi: 10.1007/s00467-015-3118-22595324736.
LinYJ
HwangB
LeePC
YangLY
Laura MengCC
2008Mid-aortic syndrome: a case report and review of the literatureInternational Journal of Cardiology1233348352doi: 10.1016/j.ijcard.2006.11.1671732161937.
AkhtarMI
HamidM
AmanullahM
KhanM
Shahabuddin
KhanMA
2007Mid aortic syndrome correction: anaesthetic considerations and managementJPMA the Journal of the Pakistan Medical Association57115635651806252438.
GospinTA
KnudsonJD
PetitCJ
2012Neonatal midaortic syndrome and renal artery atresia presenting as malignant hypertensionPediatric Cardiology335869871doi: 10.1007/s00246-012-0299-3172244738339.
HetzerR
AbsiD
MieraO
SolowjowaN
SchulzA
JavierMF
Delmo WalterEM
2013Extraanatomic bypass technique for the treatment of midaortic syndrome in childrenAnnals of Thoracic Surgery961183189doi: 10.1016/j.athoracsur.2013.03.0252368416140.
PouliasGE
SkoutasB
DoundoulakisN
PrombonasE
HaddadH
PapaioannouK
KourtisK
1990The mid-aortic dysplastic syndrome. Surgical considerations with a 2 to 18 year follow-up and selective histopathological studyEuropean Journal of Vascular Surgery417582doi: 10.1016/s0950-821x(05)80042-8232342341.
SaxenaA
KothariSS
SharmaS
JunejaR
SrivastavaS
2000Percutaneous transluminal angioplasty of the aorta in children with nonspecific aortoarteritis: acute and follow-up results with special emphasis on left ventricular functionCatheterization and Cardiovascular Interventions494419424doi: 10.1002/(sici)1522-726x(200004)49:4<419::aid-ccd15>3.0.co;2-i
1075176910.1002/(sici)1522-726x(200004)49:4<419::aid-ccd15>3.0.co;2-i42.
KimHB
VakiliK
Ramos-GonzalezGJ
SteinDR
FergusonMA
PorrasD
LockJE
ChaudryG
AlomariA
FishmanSJ
2018Tissue expander-stimulated lengthening of arteries for the treatment of midaortic syndrome in childrenJournal of Vascular Surgery67616641672doi: 10.1016/j.jvs.2017.09.0522934243043.
SoumerK
DerbelB
BenomraneS
ElleuchN
KalfatT
BenmradM
GhediraF
DenguirR
KhayatiA
2015La coarctation de l’aorte thoracique descendante: MID-aortic syndrome. À propos de trois casJournal Des Maladies Vasculaires4014248doi: 10.1016/j.jmv.2014.12.0022563164244.
FlynnJT
KaelberDC
Baker-SmithCM
2017Subcommittee on screening and management of high blood pressure in children. Clinical practice guideline for screening and management of high blood pressure in children and adolescentsPediatrics1403doi: 10.1542/peds.2017-19042882737745.
HirschJS
HongS
2019The demystification of secondary hypertension: diagnostic strategies and treatment algorithmsCurrent Treatment Options in Cardiovascular Medicine211290doi: 10.1007/s11936-019-0790-83182306746.
YanL
LiHY
YeXJ
XuRQ
ChenXY
2019Doppler ultrasonographic and clinical features of middle aortic syndromeJournal of Clinical Ultrasound4712226doi: 10.1002/jcu.226343031859347.
MorselliF
KarunanithyN
ChowienczykPJ
FacontiL
2020Do we need more vascular imaging for the screening of secondary hypertension? Mid-aortic syndrome in a young male adultJournal of Human Hypertension349668670doi: 10.1038/s41371-020-0305-93202991248.
PatelPA
CahillAM
2021Renovascular hypertension in childrenCVIR Endovascular41doi: 10.1186/s42155-020-00176-5PMC77909923341110549.
ChalmersRTA
DhadwalA
DealJE
SeverPS
2000The surgical management of renovascular hypertension in children and young adultsEuropean Journal of Vascular and Endovascular Surgery194400405doi: 10.1053/ejvs.1999.10201080137450.
DayE
StojanovicJ
KarunanthyN
QureshiS
ReidyJ
SinhaMD
2015Middle aortic syndrome—an 8-year story of pills, pretty balloons and strutsPediatric Nephrology30813611365doi: 10.1007/s00467-015-3118-22595324751.
AgrawalH
MoodieD
QureshiAM
AcostaAA
HernandezJA
BraunMC
JustinoH
2018Interventions in children with renovascular hypertension: a 27-year retrospective single-center experienceCongenital Heart Disease133349356doi: 10.1111/chd.126082963583852.
KimHB
LeeEJ
VakiliK
SteinDR
FergusonMA
PorrasD
LockJE
Fynn-ThompsonF
FishmanSJ
2018Mesenteric artery growth improves circulation (MAGIC) in midaortic syndromeAnnals of Surgery2676e109–e111doi: 10.1097/SLA.00000000000025402899186953.
VieringDHHM
ChanMMY
HoogenboomL
IancuD
De BaaijJHF
TullusK
KletaR
BockenhauerD
2020Genetics of renovascular hypertension in childrenJournal of Hypertension381019641970doi: 10.1097/HJH.00000000000024913289027254.
PappaccogliM
Di MonacoS
Warchoł-CelińskaE
LorthioirA
AmarL
AparicioLS
BeauloyeC
BrunoRM
ChenuP
De LeeuwP
De BackerT
DelmotteP
DikaZ
GordinD
HeutenH
IwashimaY
KrzesinskiJM
KroonAA
MazzolaiL
PochE
SarafidisP
SeinturierC
SpieringW
ToubianaL
Van der NiepenP
Van TwistD
VisonàA
WautrechtJC
WitowiczH
XuJ
PrejbiszA
JanuszewiczA
AziziM
PersuA
2021European/International FMD Registry and Initiative (FEIRI), and the Working Group ‘Hypertension and the Kidney’ of the European Society of Hypertension (ESH). The European/International Fibromuscular Dysplasia Registry and Initiative (FEIRI)-clinical phenotypes and their predictors based on a cohort of 1000 patientsCardiovascular Research1173950959doi: 10.1093/cvr/cvaa10232282921
